# Cognition in soccer and futsal: evidence of validity of a 4-instrument protocol to assess executive functioning among women athletes

**DOI:** 10.1186/s40359-023-01464-0

**Published:** 2023-12-09

**Authors:** Alberto Filgueiras, Matthew Stults-Kolehmainen, Gislane Melo, Richard Keegan

**Affiliations:** 1 Psychological Sciences, University of Gloucestershire, School of Natural, Social and Sport Sciences, QU214, Francis Close Hall Campus, Swindon Road, Gloucestershire, Cheltenham GL53 7JX UK; 2 Teacher’s College, University of Columbia, New York, USA; 3 Department of Bariatric Surgery, Yale New-Haven Hospital, New-Haven, USA; 4 Department of Physical Education, Universidade Catolica de Brasilia, Brasilia, Brazil; 5 School of Sport and Exercise Sciences, University of Canberra, Canberra, Australia

**Keywords:** Sport psychology, Executive functions, Cognition, Sport expertise, Psychometrics

## Abstract

Evidence suggests that success in sports, especially soccer and futsal are linked to higher levels of executive functioning. Still, the literature does not present a homogeneous set of instruments to measure executive functions, which leads to large variability in results. In this paper, we assembled four already recognised measures to propose a valid 4-instrument protocol to assess executive functions among soccer and futsal athletes. We conducted two studies to validate the proposed protocol. We addressed known-groups validity and latent structure in Study 1 for data collected on 105 female soccer and futsal athletes from elite and lower-division clubs. Findings pointed to partial validity of the protocol - with working memory and inhibition showing the best results. For Study 2, we used performance data from 51 elite female soccer players collected throughout a season of the first division league to assess predictive validity. Our protocol was able to partially replicate previous findings and added new insights on how working memory, processing speed and higher-level executive functions might play different roles for goalscoring and assist-making skills. Specifically, study 1 did not find a significant difference between elite and lower-division athletes in higher-order executive functions as in previous studies, but it did find on visual working memory and inhibitory control which weights towards higher demands of core executive functions. On the other hand, study 2 yielded significant results for processing speed and visual working memory to predict assists among elite soccer players, but not inhibitory control as previous findings suggested. Regardless, the proposed 4-instrument protocol showed adequate criterion and structural validity in both studies.

## Introduction

Soccer is a chaotic and highly dynamic sport that requires a complex combination of physical, technical, skill, tactical, emotional, and cognitive factors for a team to maximise performance [1, 2]. For several decades, researchers have put great effort into understanding the impact of physical, technical and skill factors on soccer performance [3, 4]. Most recently, evidence suggests that tactical-related measures, such as tactical decision and anticipation, are also associated with team performance in soccer [5–7].

Psychosocial factors have been studied in the sports sciences for many years; however, only in 2017 a meta-analysis concluded that these factors are both pivotal and can be intervened upon to improve sport performance [8], for example, imagery showed an effect size ranging from .26 [9] to .68 [10] and self-talk also showed a moderate effect size of .48 [11]. Emotional, personality and perceptual aspects of soccer are well studied by the scientific literature [12, 13]; yet, only in the past two decades has the relationship between cognitive factors (especially executive function (EF)) and soccer performance been investigated [14–18].

Research in executive functions and sports have been bringing heterogenous results. For instance, Vestberg et al. [16] investigated the difference of EF as measured by the Design Fluency test (a test that requires the subject to draw different patterns from the same stimuli) among soccer players who played in first, second and third division clubs in Sweden. Test performance was significantly higher in first division soccer players when compared to lower divisions. However, they assessed only higher-level EF, defined by Diamond [19] as a product of the three core EFs, named Working Memory (WM; i.e., the ability to hold and manipulate novel information in mind), Inhibitory Control (IC; i.e., the ability to inhibit distractions and focus attention on the task ahead) and Cognitive Flexibility (CF; i.e., the ability of shifting the focus of attention and taking a different perspective to solve a problem more efficiently). This means that researcher had no way to understand the real role of core and higher-order EFs in different types of sports.

Gonzaga and colleagues [5] found that decision-making assessed by the Iowa Gambling Task (IGT) is associated with tactical behaviour among young athletes, but, again, it was unclear whether core EFs that support decision-making have different roles in tactical behaviour or sport performance. To solve this puzzle, Vestberg et al. [17] investigated the role of core EFs and their product, the higher-level EFs among footballers. Core EFs were assessed using the Stroop test (IC), n-Back task (WM) and trail-making test (CF), whereas higher-level EF was assessed by the Design Fluency test (DFT). The results showed that youth soccer players perform better than the norm in WM and DFT. Their findings also suggested that the average number of goals of those athletes was correlated to WM and higher-level EF. However, instrument choice tends to have an impact on results [20], for instance it is unclear, though, whether they would have yielded the same results if they utilised a visual span task instead of a n-Back task.

The differences between elite and lower-division athletes in EFs has been investigated in several studies [14, 16–18, 21–26]. It seems that executive functioning may vary by sport type among elite and recreational athletes [23, 27]. Nevertheless, results were quite diverse among these studies in terms of core and higher-order EFs. For instance, among F1 pilots, working memory and cognitive flexibility seem to be associated with driving skills [21], among ultra-marathon runners, inhibitory control, but not working memory is associated with levels of competitiveness [28], among table-tennis athletes, all EFs were linked to levels of competitiveness [22], among young tennis players, higher-order EFs were linked to future performance, but the study did not investigate core-EFs [25], among shooters, was inhibition significantly associated with performance, but no other EF was investigated [29].

**Fig. 1:**
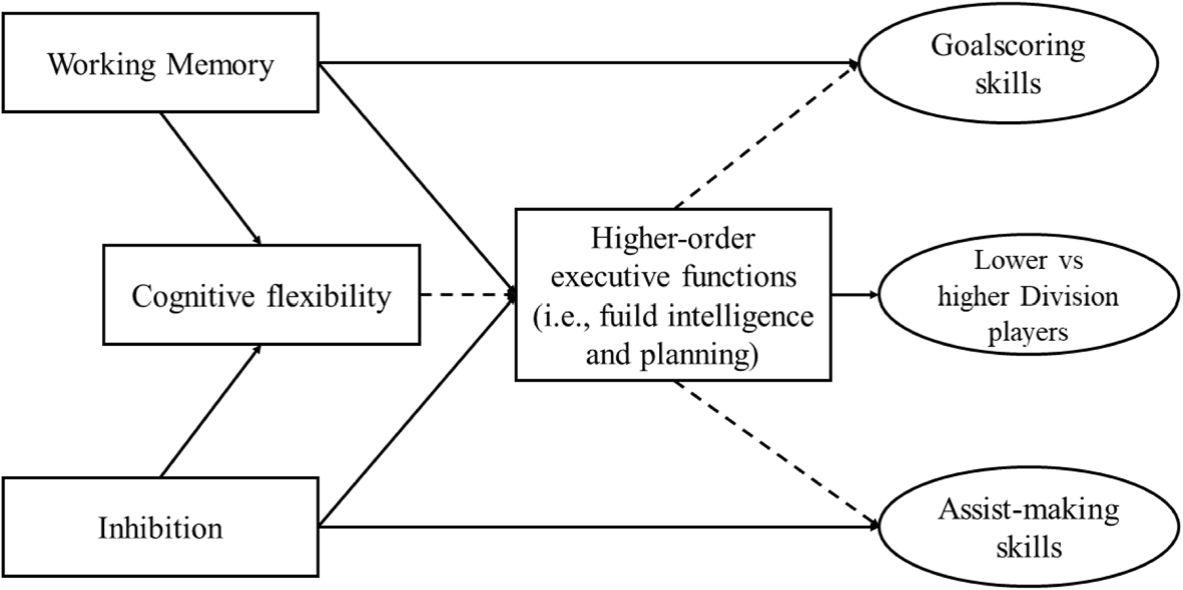
Relationship between executive functions and soccer skills. Note: Solid lines entail direct association, whereas dashed lines comprise relationships that have lower-order cognitive functions accounting for a significant part of their variance as well

The diversity of results remained among soccer studies. For instance, Sakamoto et al. [14] found differences between under-12 soccer players who got accepted in elite clubs’ youth academies and those who were not accepted. Huijgen et al. [24] found that inhibitory control and cognitive flexibility were linked to levels of competitiveness among under-18 soccer players, but neither working memory nor higher-order EF yielded significant results. In contrast, Verstberg et al. [17] found the exact opposite of Heijgen et al’s [24] study, finding significant differences in working memory and higher-order EFs, but not in inhibition and cognitive flexibility. Current research can give some idea on the relationship between athletic performance and EFs, but it still quite diverse and unclear how these results can increase homogeneity. Figure 1 depicts a diagram to offer a better view of the relationship between core and higher-order EF, and their association with soccer variables found so far.

One of the main problems for the diversity of results is the diversity of instruments utilised in this research. A recent meta-analysis gathered these findings and reached the conclusion that, even though there are significant differences between elite athletes, amateur athletes, and non-athletic samples in executive functioning, the large variety and heterogeneity of protocols to assess EFs renders it difficult to compare studies [30].

Utilising uniform and standardised protocols for psychological assessments provides several advantages. First, psychology, sport and exercise sciences are both well-known for difficulties in reproducibility, and homogeneity in assessment protocols may lead to more replicable results [31, 32]. Second, uniformity allows clearer comparisons between countries and cultures, as long as the protocol is also cross-culturally adapted or validated [33]. Assessing EF in sport is difficult due to lengthy protocols, expense, and lack of standardisation. We attempted to create a protocol that would be relatively easily administered, acceptable to athletes and coaches and inexpensive. This paper attempts to provide an initial validation of the protocol but does not attempt to compare it to more elaborate and expensive protocols, which could be done at later times. For instance, the Delis-Kaplan Executive Function System [34] adopted in studies conducted by Vestberg et al. [16, 17] can cost up to £1124.00, equivalent to US$1356.61 [35], and it is neither adapted nor validated to most countries in Latin America, Asia or Africa, which means that using those standardised instruments in soccer research may lead to narrowing the view of the phenomenon to a set of countries that uses the same instrument, neglecting the others. This lack of diversity in psychological research, especially sport psychology, is highlighted by Hassmén, Keegan and Piggott [36] who point out how it can be a problem to general understanding of sport phenomena.

Summarising, here are the current problems literature faces in executive functions and sport performance research: lack of homogeneity in assessment measures, high cost for researcher outside mainstream rich countries and a clear replication crisis. A high degree of heterogeneity in the literature has, up to this time, prevented careful comparison of EF data with systematic reviews and meta-analyses in the area of soccer and futsal research. In this manuscript we propose a solution, we assembled already existing instruments to build a cost-effective and valid 4-instrument protocol for measuring core and higher-level EFs among women soccer and futsal players. We did not create new instruments because we understand that current measures are good enough to capture statistical differences between levels of competitiveness and sport performance; accordingly, to establish a battery to test EFs among athletes seems to be a good proposition to avoid future research to collect in a single core EF (e.g., 25, 27), thus increasing comparison and replicability.

In order to accomplish our goal, we designed two studies using the same protocol for data collection. Study 1 validates this 4-measure protocol using a Path Analysis, which is a method of Structural Equation Modelling (SEM), to find the relationship between instruments—convergent validity—and investigates evidence of discriminative or known-groups validity by comparing levels of specialisation (elite vs lower-division) and types of sports (soccer vs futsal). Whereas Study 2 provides evidence of predictive validity adopting multiple linear regression with data collected from female elite soccer players. We will attempt to predict number of goals scored and assists made within a season.

### Study 1

#### Methods

##### Participants

Athletes from soccer and futsal (indoor soccer) were recruited through institutional partnerships between Catholic University of Brasilia, Rio de Janeiro State University and local sport clubs leading to a convenience sample of 105 volunteers. The need for this convenience sampling method is justified due to the rarity of elite athletes in the general population. Among those participants, 16 were elite futsal athletes (15.2%), and 20 were futsal athletes from lower-division clubs (19.1%). Twenty-one volunteers (20.0%) were elite soccer players, whereas 48 (45.7%) were lower-division football players. All participants were women.

We adopted the following criteria to consider athletes as elite: at least 5 days of practice per week, earning a salary from the club, a sponsor, or both, competing at the international level or in the first national division [37]. All lower-division athletes were professional (i.e., earning a salary from a club) but competing in lower divisions of national or regional championships. Participants received both oral and written descriptions of the research goals and signed the Term of Consent before participation.

##### Ethical compliance section

This set of studies complied with the 1964 Declaration of Helsinki, until its most recently signed amendment at the General Assembly in October 2013. Accordingly, since data collection was conducted in Brazil, we also proceeded in accordance with the National Council of Health from the Brazilian Ministry of Health. The research project that originated this manuscript was submitted to the Rio de Janeiro State University Ethics Committee for Human and Social Sciences under number CAAE16542619.2.0000.5282 before data collection. After consideration, it was approved by the consubstantiated report #2.990.037 in March 2020. All data collection and research procedures were approved by the abovementioned committee. Participants receive a briefing of the investigation, and they all signed an informed consent prior to their participation. Due to risk of confidentiality and anonymity breach due to the public nature of soccer and futsal performance statistics, only behavioural data is available upon reasonable request.

##### Procedure

We began the recruitment of participants in sport clubs in the cities of Brasilia and Rio de Janeiro after we received the approval of the research project. Clubs involved in other research projects with Catholic University of Brasilia and Rio de Janeiro State University were contacted to solicit interest for participation in the present study.

Data collection occurred in the clubs’ facilities. Athletes were informed individually of the research goals and procedures. Those who agreed to participate signed the Term of Consent and were presented with the instruments in the following order: demographic questionnaire, Stroop test (*inhibiting*), Trail-Making Test (*shifting*), Corsi-Blocks (*visuospatial span*) and Five-point task (*higher-level executive functioning*). Data was collected by one of the authors (AF) and two trained psychology undergraduate interns with 6 months of training in how to approach, present and collect data in a standardised manner. Participants took about 20 min to complete their participation.

##### Instruments

Demographic questionnaire – This was a simple self-reported questionnaire with six open-ended questions: (i) age, (ii) education, (iii) height, (iv) weight, (v) years of practice of this sport, (vi) number of days of practice per week. Additionally, three multiple-choice questions: (i) type of sport, (ii) level of competitiveness, and (iii) whether the participant practiced another sport or not.

Stroop Test – This is an inhibiting task that involves inhibiting a prepotent behaviour and replacing it for the right one. We adopted the Victoria Stroop Test [38] that comprises three blocks of testing with 24 trials each: (i) painted rectangles, (ii) congruent condition and (iii) incongruent condition. The participant needs to name colours according to the stimuli. Block 1 requires one to name the colours of rectangles painted in four different colours: yellow, blue, green and red. Block 2 needs one to name colours of words; words are the name of the colours they are tinted, for example, blue is written in a blue tint. Block 3 demands one to name colours of words; differently from block 2, this block has colour names written in different colours that they are tinted. For example, green is written in a blue tint. Measures of the Victoria Stroop Test are time of execution and number of errors for each block. The Stroop effect is calculated by reducing block 1’s time of execution from block 3’s. We adopted the Brazilian-standardised version [39].

Trail-Making Test – This is a shifting task that comprises two blocks: (1) processing speed and (2) alternating stimuli [40]. Block 1 (TMT-A) requires one to link numbers 1 to 25 scrambled on a sheet without removing the pen from the paper. Block 2 (TMT-B) requires one to link numbers 1 to 13 and letters A to L by alternating numbers and letters (i.e., 1-A-2-B-3-C and so forth). The number of errors and execution time are collected. We adopted the Brazilian-standardised version [39].

Corsi-Blocks – This is a visuospatial span task [41] that assesses updating. Participants need to tap the same blocks as the researcher in the same order for forward span and from the last to the first in the backward span. The largest number of blocks the participant can keep in mind is the measure of their span. We utilised the adult norms for the Corsi-Blocks forward span test [42].

Five-points test – The five-points test is a design fluency assessment [43] that requires the subject to draw different patterns from the same stimulus, similar to the five dots side of six-sided dice. The five-points test is considered a higher-order EF measure due to its combination of WM and CF and the component of creativity involved in the task [44]. The final score is the sum of novel patterns minus the number of repeated patterns.

##### Data analysis

Descriptive statistics were conducted for the whole sample and separately by groups. We calculated means and standard deviations for continuous and ordinal data. Frequency and percentage were used for nominal data. For each variable, we also calculated a 95% confidence interval with lower and higher bands. To ensure enough variance to conduct inferential statistical analyses, we adopted the recommendation of Hair et al. [45] of more than 100 participants. Nevertheless, because we would conduct comparisons using ANOVA with small groups of participants, we trusted the studies from Blanca-Mena et al. [46, 47], Nguyen [48] and Wang [49] who suggested that ANOVA, and the studies of Lorenco et al. [50] and Knief and Forstmeier [51] who argued that multiple linear regressions are robust enough for non-normal distributions and possible outliers.

A two-way ANOVA (level of competitiveness x type of sport) was performed to understand the differences between the two independent variables. Level of competitiveness had two levels (elite x lower division), whereas type of sport also had two levels (soccer x futsal). To measure type-2 errors, we included the power-of-the-test (1-*β*). Additionally, the effect-size (*η*^2^) was calculated. To make sure that neither age nor years of experience had an effect in our results, two independent two-way ANOVA were also performed using age and years of practice as dependent variables. In case of any significant differences in these ANOVAs, we performed additional ANCOVAs to exclude possible data bias. Post-hoc analyses were performed adopting the Least Significant Difference (LSD) and independent samples t-test (Cohen’s *d* was the measure of effect-size in those cases).

Path analysis is a method of modelling empirical data based on SEM. Using the Maximum Likelihood method of estimation from Miyake et al. [52] provided information about the association between EFs. Inhibiting (i.e., Stroop test), working memory (i.e., Corsi blocks) and cognitive flexibility (i.e., trail making test, trial B) should predict higher-level executive functioning (i.e., five-points test). We calculated regression coefficients (beta weights) and significance (*p-*value). Analyses were performed in the software R with the psych and lavaan packages. Power of the test (1-*β*) and effect-size (*η*^2^) were calculated using G-Power.

## Results

Normality of continuous variables was confirmed by skewness and kurtosis. Average of age was 23.59 (SD = 4.46), years of practice was 12.16 (SD = 3.10), number of days of practice per week 5.60 (SD = 1.25), averaged height 166 cm (SD = 6.33 cm), and weight was 61.48 kg (SD = 7.54 kg). Among the participants who were lower-division futsal players, 7 (35.0%) had completed high school, whereas 13 (65.0%) completed college. The elite futsal players were divided into those with a high school degree (4, 25.0%) and those with a college degree (12, 75.0%). Amid the lower-division football/soccer players, 26 (54.2%) reported having a high school degree and 22 (45.8%) had a college degree. Among the elite soccer/football players, 13 (61.9%) had completed high school, while 8 (38.1%) had a college degree. A chi-square statistic showed no significant concentration of any type of education between groups [χ2 = .238; *p* = .626]. Descriptive statistics per group are presented in Table 1.

**Table 1:** Descriptive statistics divided by type of sport (futsal and soccer) and level of competitiveness (lower division and elite)

Sport	Level	Variable		Statistics			95% CI	
				M	SD	SEM	Lower	Higher
Futsal	Lower Division (*N* = 20)	Age		21.50	3.05	.68	20.26	22.82
		Years of Practice		12.55	2.14	.49	11.64	13.50
		Days of Practice per week		5.80	.89	.20	5.41	6.19
		Height		169.95	7.05	1.63	162.80	169.20
		Weight		60.05	6.95	1.55	56.86	63.14
		BMI		21.79%	2.07%	0.46%	20.86%	22.66%
		Five-points test		18.64	6.32	1.38	13.69	23.58
		Stroop Test		8.95	.94	.21	8.52	9.37
		TMT-A	Time	50.25	11.07	2.53	45.27	55.23
			Errors	.15	.49	.11	.00	.39
		TMT-B	Time	62.90	11.97	2.77	57.72	68.42
			Errors	.30	.66	.14	.05	.61
		Corsi Blocks		4.40	1.23	.29	3.85	4.94
	Elite (*N* = 16)	Age		23.32	4.41	.64	21.14	23.58
		Years of Practice		11.94	2.11	.36	11.62	13.00
		Days of Practice per week		5.50	.52	.13	5.41	5.91
		Height		165.25	3.68	1.00	163.71	167.66
		Weight		59.25	9.60	1.38	57.13	52.51
		BMI		21.71%	3.60%	0.47%	20.92%	22.74%
		Five-points test		17.41	10.60	2.74	9.55	25.27
		Stroop Test		10.25	.68	.18	9.17	9.87
		TMT-A	Time	41.63	14.62	2.25	42.12	51.19
			Errors	.13	.50	.08	.00	.30
		TMT-B	Time	54.75	10.35	1.98	55.43	63.42
			Errors	.19	.54	.10	.07	.45
		Corsi Blocks		5.35	1.83	.29	4.60	5.73
Soccer	Lower Division (*N* = 48)	Age		24.27	4.60	.66	23.83	26.60
		Years of Practice		11.85	3.70	.52	10.78	12.84
		Days of Practice per week		6.46	.50	.07	6.31	6.60
		Height		166.21	6.29	.92	164.42	168.04
		Weight		60.67	6.22	.92	58.77	62.62
		BMI		21.97%	2.05%	0.30%	21.37%	22.56%
		Five-points test		15.43	4.69	1.77	12.51	18.34
		Stroop Test		9.29	.77	.11	9.06	9.51
		TMT-A	Time	49.48	14.40	2.13	45.43	53.79
			Errors	.15	.46	.07	.03	.29
		TMT-B	Time	64.00	15.60	2.30	59.20	68.31
			Errors	.17	.48	.07	.04	.31
		Corsi Blocks		5.71	1.50	.21	5.59	6.40
	Elite (*N* = 21)	Age		21.95	4.03	.89	20.21	23.69
		Years of Practice		12.67	3.07	.67	11.40	14.00
		Days of Practice per week		3.52	.51	.11	3.32	3.74
		Height		167.67	7.39	1.55	164.77	170.99
		Weight		66.38	7.05	1.51	63.50	69.40
		BMI		22.57%	1.37%	0.30%	21.96%	24.15%
		Five-points test		18.67	7.91	1.01	25.57	11.78
		Stroop Test		10.05	.74	.16	9.73	10.36
		TMT-A	Time	42.38	13.20	2.87	37.12	48.44
			Errors	.14	.48	.10	.00	.38
		TMT-B	Time	59.05	11.17	2.42	54.46	63.76
			Errors	.19	.51	.11	.00	.45
		Corsi Blocks		6.00	1.58	.35	5.22	6.60

TMT-A stands for Trail Making Test, trial A, whereas TMT-B stands for Trail Making Test, trial B

Demographic variables were: age, years of practice, days of practice per week, height, and weight; whereas higher-level executive functioning was measured by the Five-points test score, inhibition by the Stroop test score, processing speed by the TMT-A, cognitive flexibility by TMT-B and visuospatial working memory by the Corsi blocks performance

**Fig. 2:**
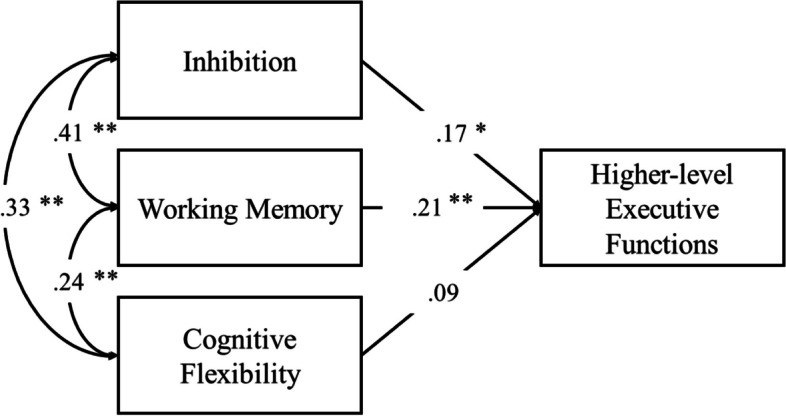
The Path Analysis presenting the regression coefficients of executive functions assessed by the Stroop test (inhibition), Corsi blocks (visuospatial working memory), Trail Making test, trial B (cognitive flexibility) and the Five-points test (higher-level executive functioning). Note: * *p* < .05; ** *p* < .001

Regarding the Path Analysis, all associations between core and higher-order EFs were significant and yielded low-to-moderate beta weights, with the exception of cognitive flexibility and higher-level executive functioning, which had a non-significant association (*β* = .09; *p* = .205). The highest association was between inhibition and working memory (*β* = .41; *p* < .001), and the lowest significant association was between inhibition and higher-order EF (*β* = .17; *p* = .015). Figure 2 depicts the Path Analysis with its respective beta weights.

To ensure that neither age nor experience (measured in years of practice) were different within or between groups, we performed two two-way ANOVAs (level of competitiveness x type of sport). Age ANOVA was significant [*F*(3,101) = 5.279; *p* = .002; *η*^2^ = .136; power = .921], however no differences between levels of competitiveness [*F*(1,104) = .708; *p* = .402; *η*^2^ = .018; power = .266] nor between types of sports [*F*(1,104) = 1.813; *p* = .181; *η*^2^ = .007; power = .133] were found. In fact, age only differed statistically between lower-division futsal and soccer players [*t*(66) = 3.362; *p* = .001; *d* = .966; power = .951], soccer players were significantly older than futsal players in lower-division clubs. Experience was non-significant [*F*(3,101) = .468; *p* = .705; *η*^2^ = .014; power = .142]. Based on these results, we calculated two-way ANCOVAs using age as a co-variable beyond the main-effect comparisons to determine whether this factor had any influence on ANOVAs’ results.

The standardised Victoria Stroop Test showed significant differences [*F*(3,101) = 12.525; *p* < .001; *η*^2^ = .271; power = .999] with statistically different main-effect for level of competitiveness [*F*(1,104) = 37.557; *p* < .001; *η*^2^ = .271; power = .999]. However, we found no main-effect for sport type [*F*(1,104) = .172; *p* = .679; *η*^2^ = .002; power = .070] and no interaction [*F*(1,104) = 2.630; *p* = .108; *η*^2^ = .025; power = .362]. ANCOVA with age as co-variable yielded the same results with no statistical differences in age [*F*(1,104) = 3.670; *p* = 0.051; *η*^2^ = .038; power = .505]; however, it was close to the adopted threshold of *p* < .05, which means that the results could be partially explained by the age difference. On the other hand, instead of decreasing the main-effect of our original two-way ANOVA, the ANCOVA strengthened the statistical difference for the main-effect of competitiveness level [*F*(1,104) = 40.480; *p* < .001; *η*^2^ = .288; power = .999]. Additionally, the effect-size of sport type decreased [*F*(1,104) = .023; *p* = .879; *η*^2^ < .001; power = .023], and the same happened with the interaction [*F*(1,104) = .648; *p* = .303; *η*^2^ = .011; power = .177]. Thus, using age as a co-variable increased the main-effect of level of competitiveness rather than weakened it. Elite players yielded higher scores than lower division players in the Stroop Test; however, there was no difference between soccer and futsal players.

The Trail Making Test - version A (TMT-A) showed no significant results [*F*(3,104) = 2.542; *p* = .061; *η*^2^ = .070; power = .612]. The TMT-A ANCOVA for age kept the same non-statistical significance [*F*(4,100) = 2.168; *p* = .078; *η*^2^ = .080; power = .621]. The Trail Making Test - version B (TMT-B) similarly presented no significant differences neither in the two-way ANOVA [*F*(3,101) = 2.199; *p* = .093; *η*^2^ = .061; power = .544] nor the age ANCOVA [*F*(4,100) = 1.896; *p* = .117; *η*^2^ = .070; power = .556].

The Corsi Blocks test yielded significant results in the two-way ANOVA [*F*(3,101) = 5.728; *p* = .001; *η*^2^ = .145; power = .942]. The main effect for sport type was statistically different [*F*(1,104) = 5.262; *p* = .024; *η*^2^ = .050; power = .623]. To the same extent, level of competitiveness was significantly different [*F*(1,104) = 5.561; *p* = .020; *η*^2^ = .052; power = .646] as was the interaction [*F*(1,104) = 6.699; *p* = .011; *η*^2^ = .062; power = .727]. The ANCOVA with age as covariate showed similar results, [*F*(4,100) = 4.354; *p* = .003; *η*^2^ = .148; power = .923]. The was a main effect for sport type [*F*(1,104) = 5.494; *p* = .021; *η*^2^ = .0.52; power = .641], and level of competitiveness [*F*(1,104) = 5.260; *p* = .024; *η*^2^ = .050, power = .622] but no difference for age [*F*(1,104) = .344; *p* = .559; *η*^2^ = .003; power = .089]. The interaction remained significant [*F*(1,104) = 6.979; *p* = .010; *η*^2^ = .065; power = .744]. Soccer players showed higher scores in Corsi Blocks than futsal players, whereas lower division futsal players presented lower scores than elite futsal players.

Finally, the Five-points test retrieved non-significant results in the two-way ANOVA [*F*(3,101) = 1.919; *p* = .136; *η*^2^ = .019; power = .700]. The Five-points test ANCOVA using age as a covariate kept non-significant differences [*F*(4,100) = 2.253; *p* = .093; *η*^2^ = .023; power = .654].

### Study 2

#### Method

##### Participants

Volunteers for the present study consisted of 51 female elite soccer players from three first division soccer clubs in the Brazilian Soccer League (Série A) who did not participate of study 1. These are among the most talented soccer players in the world. No goalkeepers were included due to the characteristics of the position. Only players who player at least three matches in the season were included due to statistical purposes. All athletes signed the Term of Consent and were assessed at the beginning of the season before sanctioned matches commenced.

##### Procedure

At the beginning of the competitive season (January and February), the first author approached physicians and coaches of six Brazilian clubs in the first division (highest level) to recruit their female professional players for participation. Three clubs agreed and recruitment occurred. The athletes who volunteered had their cognitive and demographic (age and education) information collected by the first author on a pre-scheduled date before the beginning of the Brazilian Soccer League. Data was collected in a quiet environment provided by the clubs. This study was part of the same project as study 1 and was approved by the Rio de Janeiro State University Ethical Committee under the consubstantiated report #2.990.037 in March 2020.

##### Instruments

Our 4-instrument protocol for EF assessment was the same as study 1: Stroop test for IC, Corsi blocks for vWM, Trail Making test, trial B (TMT-B) for CF and the Five-points test for higher-order EFs. Regarding soccer statistics, we used the platform *Footstats* [53]. *Footstats* is a web-based server that contains performance statistics of players from the first and second division teams in the Brazilian League (*Serie A* and *Serie B*, respectively). Among the several options, those statistics linked to shots and passing were chosen due to the dependent variables of the present study: goals and assists. The six soccer stats chosen were: 1) average total passes given per match, 2) average correct passes given per match, 3) average total shots made on the goal per match, 4) average successful shots on the goal per match (i.e., goalkeeper defences and goals), 5) average dribble attempts per match, and 6) average successful dribbles given per match. The player’s field position (attacker, midfielder, and defender) was also included in the analysis. Finally, the total number of matches played by the athlete (minimum of 4 matches/year and maximum of 58 matches/year), the total number of goals in the season per player and the total number of assists in the season per player were extracted from the platform.

##### Data analysis

After the end of the same season, data from performance statistics were extracted from the *Footstats* platform [53] and combined with cognitive and demographic data from the season start. Descriptive statistics were calculated based on Stevens [54] recommendation: for categorical data, number (frequency) and percentage were used, whereas for continuous data, arithmetic average (mean) and standard deviation (SD) were adopted.

Two separate mixed-effects model regressions were performed with the dependent variables (DV) of total number of assists and goals across the season. Independent variables (IV) entered into the regressions were: the five outcomes from our 4-instrument EF measure [i.e., (i) Corsi blocks score, Trail Making Test (ii) trial A and (iii) trial B, (iv) Stroop test and the (v) five-points test], the six soccer stats [i.e., (i) average total passes given per match—total passes, (ii) average correct passes given per match—correct passes, (iii) average total shots made on the goal per match—total shots, (iv) average successful shots on goal per match—correct shots, (v) average total dribble attempts per match—dribble attempts, and (vi) average successful dribbles given per match—successful dribbles.] And the player’s position on the field was included as the mixed-effects model, categorised by groups: attackers, midfielders, and defenders. For the purpose of this study, assists were not included in the passes stats neither were goals included in shots stats.

The stepwise method was chosen to run the regression considering a significance level of *p* < 0.05 as the inclusion criterion. Once the relevant IVs were selected in the analysis, the coefficient of determination (*r*^2^), which determines the amount of variance explained by the regression, was calculated for the whole regression, for the cognitive and soccer stats measures separately, and for the player position. The coefficient beta (*β*) and the t-test statistics (*p*-value, power of the test and effect-size) revealed the strength of each IV to predict the DV; whereas t-tests provided the level of significance for each IV in association with the DV when compared to the null-hypothesis – the constant (or intercept). Finally, we calculated an ANOVA for each regression to compare the null hypothesis (the constant) to the mixed-effects model including significant IVs. According to Blanca-Mena et al. [46, 47], and Wang et al. [49] ANOVA is sufficiently robust to hold non-normal distribution whereas Knief and Forstmeier [51] and Lourenco and colleagues [50] argue that linear modelling is robust enough to avoid type 1 errors and, more importantly, replication errors. Analyses were performed in the same computer applications of study 1.

## Results

The participants were divided by soccer field position: wingers, forwards and strikers were classified as attackers (*N* = 18). Defensive, central, and attacking midfielders were categorised as midfielders (*N* = 15). Finally, centre, sweepers, full- and wingbacks were labelled as defenders (*N* = 18). Goalkeepers were excluded from the present research. The three teams played an average of 54.7 (SD = 6.3) matches during the season including the first division of the Brazilian Soccer League, the Libertadores da America Cup (Latin American Cup) and State Championships. Table 2 depicts the descriptive statistics for players who participated in this study.

**Table 2:** Descriptive statistics of the sample considering executive functions, season soccer stats and demographics

Variable		Descriptive Statistics			95% CI	
		Mean	SD	SEM	Lower	Higher
Demographic						
Number of matches played in the season		22.20	14.56	2.04	9.68	34.72
Age		28.94	8.76	1.23	21.41	36.47
Education (in years)		11.86	4.72	.66	7.80	15.92
Soccer (season) statistics						
Total number of goals in the season		3.47	4.70	.66	.27	7.51
Total number of assists in the season		2.40	2.51	.35	.24	4.56
Total shots given per game		1.33	1.02	.14	.45	2.21
Shots in the target given per game		.35	.13	.02	.24	.46
Total passes given per game		41.87	14.10	1.97	29.74	54.00
Correct passes given per game		35.90	13.16	1.84	24.58	47.22
Total dribble attempts per game		2.88	2.26	.32	.94	4.82
Successful dribbles per game		1.72	1.45	.20	.47	2.97
Executive Functions						
Corsi Blocks		6.60	.56	.08	6.12	7.08
Trail Making Test	Time	16.83	3.56	.50	13.77	19.89
	Errors	.16	.46	.07	.04	.30
Trail Making Test	Time	28.59	9.34	1.31	20.56	36.62
	Errors	.15	.47	.07	.03	.28
Stroop test		24.83	4.84	.68	20.67	28.99
Five-points Test		19.73	6.43	.90	14.20	25.26

TMT-A stands for Trail Making Test, trial A, whereas TMT-B stands for Trail Making Test, trial B

The mixed-effects model regression used to predict the total number of goals in the season was significant: *F*(3,10) = 23.66; *p* < .001; *f*^2^ = 1.43; power = .99. Field position, Corsi blocks (vWM) and the Five-point test (higher-level EF) predicted 59% (*r*^2^ = .59) of the variance. Additionally, the field position explained 11% of the variance, whereas cognitive variables explained 34% of the variance. The remaining variance was explained by the constant (intercept). No season soccer stats were able to significantly predict number of goals.

Furthermore, the regression to predict total assists throughout the season was significant: *F*(4,9) = 12.70; *p* < .001; *f*^2^ = 3.76; power = .99. Average passes made per match, average successful dribbles per match, Corsi blocks (vWM) and trial A of the Trail Making Test (i.e., processing speed) altogether predicted 79% (*r*^2^ = .79) of the variance. Separately, performance measures (i.e., total passes and successful dribbles) explained 33% of the variance, whereas cognitive measures (i.e., vWM and processing speed) explained 49% of the variance. The constant, however, was not significant. Table 3 presents the results of the two mixed-effects regression analyses.

**Table 3:** Mixed-model regression analysis for number of goals and assists in the same season of the data collection

Variable	Mixed-model regression statistics						
	*β*	*t*-test	*p*-value	effect-size	power	*r*^2^	
Goals							.59
(Intercept)	5.76	2.56	<.01	.85	.99		
*Mixed-level*						.11	
Position in the field	1.12	2.42	<.05	.21	.91		
*Cognitive measures*						.34	
Corsi blocks	.83	2.14	<.05	.51	.99		
Five-points Test	.87	2.13	<.05	.26	.93		
Assists							.79
(Intercept)	2.13	.77	.45	.03	.20		
*Soccer statistics*						.33	
Total Passes	.09	5.19	<.01	.17	.81		
Successful Dribles	.51	4.60	<.01	.40	.99		
*Cognitve measures*						.46	
Corsi blocks	.37	6.73	<.01	.63	.99		
Trail Making Test (Trial A)	.97	2.20	<.05	.11	.60		

We calculated separate mixed-effects model regressions for number of goals in the season (goals) and number of assists in the season (assists)

## Discussion

This paper presents two studies conducted to develop a valid and cost-effective protocol to assess executive functioning among soccer athletes using four instruments that can be easily found and used by researchers. Standardised commercial instruments are not always the first option for developing and poor countries, because the standardisation process relies on cross-cultural adaptations, a significant financial investment might be needed to achieve it, and researcher from less privileged countries might not be able to have it at their disposal [30]. Translation and adaptation require effort of researchers from several countries to achieve the goal of having equivalent standards [55]. Nevertheless, it is rare to find the same standardised EF measures in many countries [30], mainly because most commercial instruments are neither published nor cost-effective for researchers.

Study 1 initially provides criterion and group validity with significant differences between levels of competitiveness (i.e., elite vs lower division female soccer players) and between types of sport (i.e., soccer vs futsal) in some, but not all tested EFs. The study of Vestberg et al. [16] found statistical difference in higher EFs among male soccer players from the first and the fourth division of the Swedish league, whereas our study did not find the same results regarding higher-order EF, we did find it in vWM and inhibition. Results comparing EFs in diverse levels of competitiveness were similar [14, 17, 18, 21–26]. Which means that our study was able to reproduce previous findings in a sample of Brazilian female soccer and futsal players. The same thing happened to type of sport [56, 57]. Even though soccer and futsal players use mainly their feet and execute equivalent techniques, the size of the pitch and the indoor vs outdoor variables might lead to cognitive differences, since evidence was already found to support physiological and technical distinctions [1, 58]. Indeed, vWM was statistically distinct and corroborated with our hypothesis.

We added to study 1 a structural validity analysis based on Miyake et al.’s [52] evidence of a three-factor model to explain EFs: WM, inhibition, and CF. Nonetheless, we used the higher-level EF as well which led to a SEM path-analysis with three correlated variables (i.e., core EFs) and one predicted variable (i.e., higher-order EF). Our results showed good correspondence to Miyake et al.’s [52] regarding significant relationship between the three core EFs, however, CF and higher-level EF were not associated. It is important to remember that Diamond [19] suggests that CF is a subproduct of WM and inhibition, thus, in a SEM WM and inhibition could pull the regression weights loadings leaving non-significant association between CF and higher-order EF due to a moderation effect.

Results from study 1 provided partial validity to our 4-instrument protocol. Corsi blocks (vWM) and Victoria Stroop Test (inhibition) were valid in both group validity and structural validity. They showed statistical differences in type of sport and levels of competitiveness, respectively, and presented significant association between them and other EFs. However, neither Trail Making Test (CF) nor the Five-points Test (higher-level EF) retrieved significant results in study 1. The only support for validity of these instruments would be their significant relationship with vWM and inhibition. Nonetheless, they were not significantly associated between each other, which may lead to doubts regarding its use in this protocol. Since study 1 was not enough to support the use of these four measures, we developed study 2.

We designed study 2 to support further validity of our protocol based on predictive validity. Accordingly, vWM and higher-order EF, as measured by Corsi blocks and the Five-points test, respectively, significantly predicted the total number of goals made by Brazilian elite female soccer players during the season. It corroborates with previous findings from Verburgh et al. [14], and Vestberg et al. [17]. It seems that the mechanisms of decision-making while a soccer player is trying to score a goal rely strongly on vWM and higher-level EFs, even more than other technical and tactical variables such as precision or quantity of shots made throughout the season. It is a matter of technical execution combined with cognitive skills [58]. Regarding assists in the season, vWM and processing speed were able to significantly predict this variable. It is similar to the results found by Verburgh et al. [14] who found that inhibition and CF were associated to assists. Even though our study did not yield significant association between assists and inhibition, vWM has a strong attentional component and performance in the Trial A of the TMT relies strongly on processing speed, which is the foundation of CF. Altogether, the use of both the Five-points test and the TMT in the 4-instrument protocol proposed in this paper is justified by the study 2’s predictive validity results.

Altogether, results from studies 1 and 2 yielded evidence to support both the use and validity of our 4-instrument protocol to assess core and higher-level executive functioning among soccer and futsal athletes. However, we did not accomplish in totality, retrieving some results that only partially corroborated with previous literature, not entirely [14–18, 21–26, 56, 57]. Nonetheless, to have a protocol that researchers from different areas of the globe can adopt and conduct their studies might ease the issues highlighted by Kalén et al. [30], the excessive heterogeneity of measures leading to varied results that, as it happened in our studies, do not fully corroborate to each other. Another advantage of having the same 4-instrument protocol we proposed in future studies is to allow researcher from poorer countries to find and compare results with other studies, leading to an increase on variety and diversity in research on soccer and, perhaps, other sports [36]. Thus, we defend the use of this protocol in research with potential modifications to adapt it to other contexts, since it has good evidence from either group, structural or predictive validity.

The set of evidence that can be found in current literature leads to the assumption that executive functioning is pivotal in soccer performance [15–18, 30]. Yet, it is not clear how the mechanisms behind this relationship work and whether there are moderators between them. The collective work of researchers in different parts of the globe is required to better comprehend those pressing questions, however, what we have seen so far is a large amount of research on sport and exercise psychology been conducted by the same rich countries leaving other less privileged nations behind [36]. It brings the need to address methodological differences, including more cost-effective measurements and less heterogeneous approaches [30]. Our suggestion with this paper was to adopt the Corsi blocks, the Victoria Stroop test, the Trail Making test and the Five-points test to measure vWM, inhibition, CF, and higher-order EF, respectively. Our endeavour was partially successful, which leaves to future studies the challenge to use it again and to improve or adapt it to diverse contexts and types of sport beyond soccer. Once this protocol establishes itself in the literature as a valid and reliable procedure to assess executive functioning among athletes, then, it would be possible to develop a cross-cultural normative study and to provide practitioners with the tools necessary to evaluate and design interventions based on these instruments.

### Study limitations

We understand that, even though the main goal of this research was to provide a cost-effective and easy-to-do executive functioning assessment protocol for research in soccer and sport science, our studies had some limitations that need to be considered. The first is differences in age in study 1. Even though we adopted an ANCOVA to analyse age as a co-variable and retrieved no-statistical significance, maturity could explain some of the results [59]. Yet, since the age difference occurred between lower-division futsal and soccer athletes, one might have expected that cognitive flexibility would distinct between types of sports, not level of competitiveness. Thus, the age explanation is unlikely, yet possible.

The second possible limitation is education [60]. People with higher levels of education tend to show higher performance in executive functions tasks as well; however, our sample did not have any statistical differences in participants with either high school or college degrees; in fact, our volunteers only had those two degrees, and the chi-square statistics showed no specific concentration of one level of education. Thus, we had no support to claim education as a variable of explanation regarding lack of differences in cognitive flexibility in study 1 or the absence of this variable in the list of predictive validity in study 2. Regardless, even without enough evidence to support this claim, individual differences still explaining a great deal of this phenomenon.

The third limitation for our studies that also explains why we did not replicate some previous findings would be our sampling method. We collected data in a smaller convenience, thus neither representativeness nor enough variance could be ensured. Even though Knief and Forstmeier [51] argue that it is better to risk biased results due to non-normal distribution or outliers’ distortions than non-replicable findings, we need to acknowledge that generalisation of this study is compromised. Future studies should aim larger and more diverse samples to ensure further use of this 4-instrument protocol we propose.

The fourth and last limitation is that our study found a difference between groups in age in study 1 which can lead to complications in analysing and interpreting results of the Path Analysis. It could be argued that these bias in age would lead to cognitive biases and, therefore, impair our results. In the present study, we had to choose between including age as a covariable in the Path Analysis reducing its statistical power or not including and risking not to capture potential group differences [61]. Because our goal was to assess structural validity, we have chosen not to include age in the Path Analysis. However, we understand that future studies might try to avoid these issues by using more rigorous sampling methods.

## Data Availability

Due to the public nature of the performance statistics used in this study, there is a risk of breach of anonymity and confidentiality. Therefore, the ethical committee has decided that only behavioural data can be shared upon reasonable request to the corresponding author by email: albertofilgueiras@gmail.com.
